# Nano/Micromotors for Cancer Diagnosis and Therapy: Innovative Designs to Improve Biocompatibility

**DOI:** 10.3390/pharmaceutics16010044

**Published:** 2023-12-27

**Authors:** Jiahuan Zheng, Rui Huang, Zhexuan Lin, Shaoqi Chen, Kaisong Yuan

**Affiliations:** 1 Department of Chemistry, Shantou University Medical College, Shantou 515041, China; jiahuanzheng@stu.edu.cn; 2 Bio-Analytical Laboratory, Shantou University Medical College, Shantou 515041, China; 22rhuang1@stu.edu.cn (R.H.); g_zxlin@stu.edu.cn (Z.L.); 3 Department of Ultrasound, First Affiliated Hospital of Shantou University Medical College, Shantou 515041, China

**Keywords:** nano/micromotor, biocompatibility, microswarm, cancer diagnosis, cancer therapy

## Abstract

Nano/micromotors are artificial robots at the nano/microscale that are capable of transforming energy into mechanical movement. In cancer diagnosis or therapy, such “tiny robots” show great promise for targeted drug delivery, cell removal/killing, and even related biomarker sensing. Yet biocompatibility is still the most critical challenge that restricts such techniques from transitioning from the laboratory to clinical applications. In this review, we emphasize the biocompatibility aspect of nano/micromotors to show the great efforts made by researchers to promote their clinical application, mainly including non-toxic fuel propulsion (inorganic catalysts, enzyme, etc.), bio-hybrid designs, ultrasound propulsion, light-triggered propulsion, magnetic propulsion, dual propulsion, and, in particular, the cooperative swarm-based strategy for increasing therapeutic effects. Future challenges in translating nano/micromotors into real applications and the potential directions for increasing biocompatibility are also described.

## 1. Introduction

As is well-known, cancer is a serious disease that threatens human health. Today, it remains a medical challenge despite the progress achieved so far in pharmaceutical science and drug discovery [1]. Among the advanced techniques, nano/micromotors are man-made miniature devices that have arbitrary, directional, or even controllable motions to perform special tasks in microenvironments due to the energy conversion hiding behind their mechanical movement. Various types of energy, including chemical, light, magnetic, ultrasound, etc., have been employed for nano/micromotor propulsion and achieved the desired effect [2,3]. Such miniature devices can be integrated with fluorescence [4,5], electrochemistry [6,7], or the Raman signal-enhancement substrate [8,9] for biomarker detection or loaded with drugs for targeted site delivery and release [10,11,12], thus showing great potential for disease diagnosis and treatment. Indeed, a large number of nano/micromotors with different designs have already been introduced for cancer diagnosis and treatment. However, biotoxicity remains a challenge that restricts them from moving from the laboratory to clinical applications. These include (a) The toxicology of nano/micromotor components (for instance, by assessing cell viability, previous research reported that Mg/Pt Janus micromotors show a concentration-dependent toxic trend) [13]; (b) The generation of harmful byproducts [14]; (c) The use of toxic fuels at high concentrations [15]; and (d) The immune response caused by nondegradable components and the use of sperm derived from other species [16,17,18]. Recent advances show the great efforts made by researchers to improve the biocompatibility of nano/micromotors.

When using nano/micromotors for cancer treatment, the disadvantage of traditional chemical propulsion modes is the unavoidable use of toxic fuel, which hinders their future in vivo application [16,19]. The use of natural enzymes for catalytic bubble production [20,21] and magnetic fields [22,23], ultrasound waves [24,25], or light [26,27] shows great promise in providing a biocompatible method for nano/micromotor propulsion. Another major factor that leads to biotoxicity may be the materials used to produce nano/micromotors. For this reason, cell membranes have been used to coat nano/micromotors, which provide a biocompatible surface mimicking cells. In addition, living cells (such as sperm bacteria or red blood cells) have been used directly as a basic skeleton to construct nano/micromotors [28,29,30]. Apart from that, driving nano/micromotors in an in vivo microenvironment becomes much more complex since the blood flow or co-existing protein/cells/tissues can weaken their motion behavior. To address this issue, dual propulsion modes have been introduced due to their more controllable and powerful mechanical movement [31,32]. Finally, cooperation among groups of nano/micromotors (so-called microswarms) could help to achieve the delivery of larger doses of loading drugs and higher therapeutic efficacy compared to individual agents, thereby indirectly lowering the toxicity. Related details are discussed below.

For cancer biomarker detection, the integration of nano/micromotors also plays an important role in realizing high sensing efficiency. Normally, the attachment of an analyte to a related sensing substrate is the basis of effective detection. For example, surface-enhanced Raman scattering (SERS)-based sensing first needs an analyte to be adsorbed on the surface of a Raman signal-enhanced substrate (normally noble metal nanoparticles or with three-dimensional periodic structures), and fluorescence-based sensing needs fluorescence materials such as quantum dots or upconversion nanoparticles to interact with the targets. To ensure the connection between the analyte and the sensing substrate, one simple way is to mix the SERS substrate or fluorescence materials together with the analyte in a tube by simple shaking or vortex oscillation. However, for targets in some specific positions, such as the channels of a microchip, it is impossible to mix the liquid using similar operations unless we wait a long time, ranging from tens of seconds to minutes (depending on analyte concentration, electrolyte composition, diffusion distance, etc.). To allow autonomous diffusion of the analyte, using a piezoelectric micropump [33,34] or an electroosmotic pump [35,36] can help to enhance mass transfer in the channels of a microchip. However, the fabrication of such a microstructure normally requires a complex method.

The nano/micromotor described here holds promise to serve as a tool to solve the above problems. The migration of nano/micromotors enables the active capture of analyte, along with the micro-mixing effect to enhance mass transfer in a microenvironment. Therefore, the nano/micromotor is a powerful tool that can help to achieve higher efficiency in biosensing. When discussing biocompatibility with the use of nano/micromotors for cancer detection, the situation turns out to be simple compared to cancer treatment. Usually, the detection of biomarkers can be conducted by in vitro experiments that require only a small amount of blood or tissue from the body. Indeed, no direct contact between the nano/micromotors and the body occurs. However, in in situ or the real-time detection of biomarkers in some cases, such as the intracellular biosensing of a target miRNA [37], the targeting of intracellular SERS sensing [38], or medical imaging [39,40,41], as well as the biocompatibility of nano/micromotors, remains important.

**Scheme 1:**
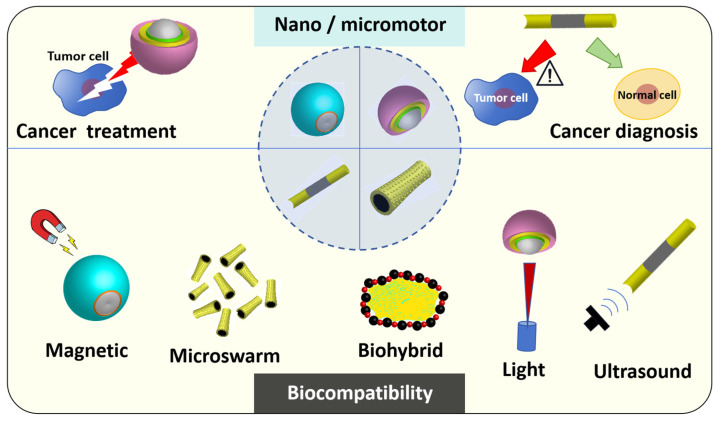
Schematic illustration showing biocompatible designs of nano/micromotors for cancer diagnosis and treatment.

In this review, we focus on recent advances in research attempts to improve the biocompatibility of nano/micromotors for cancer diagnosis and therapy. We discuss bio-friendly propulsion modes, including the biohybrid propulsion mode, ultrasound propulsion, magnetic field, light-triggered propulsion, and dual propulsion mode, as well as the cooperative swarm-based strategy (Scheme 1). Previous studies have already reviewed the development of nano/micromotors for diagnosis and therapy in cancer and infectious diseases [42]. Herein, we discuss this area from a completely different perspective: bio-friendly designs to promote their use in real applications.

## 2. Bubble Propulsion by Inorganic Catalysts

**Figure 1:**
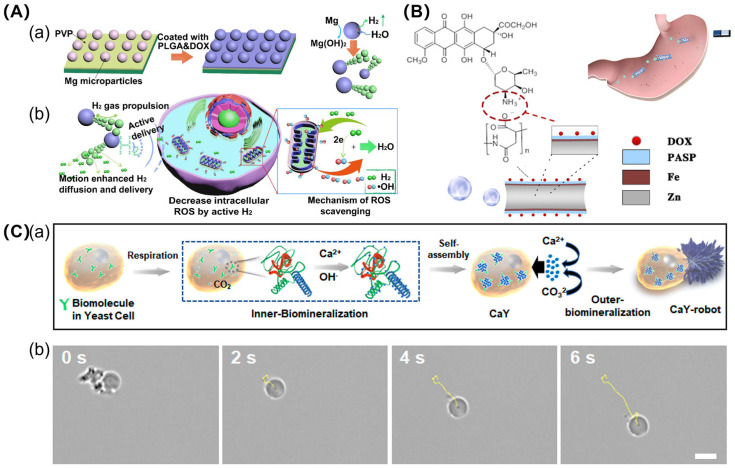
Bubble-propelled nano/micromotors using biocompatible inorganic catalysts. (**A**) Mg as biocompatible inorganic catalyst for micromotor in tumor treatment: (**a**) Schematic illustration of fabrication process of Mg-based micromotor, surface modification, and propulsion mechanism; and (**b**) Its mechanism in decreasing intracellular ROS by H_2_ delivery. Adapted from [43] with permission, copyright Elsevier Ltd., Amsterdam, The Netherlands, 2020. (**B**) Schematic structure of DOX-loaded Zn-based tubular micromotor for targeted drug delivery in the stomach. Adapted from [44] with permission, American Chemical Society, Washington, DC, USA, 2019. (**C**) CaCO_3_ as biocompatible inorganic catalyst for micromotor: (**a**) Schematic illustration of mechanism of fabrication of yeast micro/nanorobot (Cur@CaY-robot) via dual biomineralization, in which inner nano-CaCO_3_ inside yeast cells is biomineralized through cell respiration and outer nano-CaCO_3_ though axially asymmetric crystallization. (**b**) Time-lapse images showing self-propelled motion of a single Cur@CaY robot. Adapted from [45] with permission, American Chemical Society, Washington, DC, USA, 2023.

Using inorganic catalysts to generate bubbles for nano/micromotor propulsion is the most traditional and well-known way to obtain in-depth information (such as motion mechanism or different applications) in studies in this research field. A typical example is the Pt-based nano/micromotor. For biocompatibility purposes, magnesium (Mg), with biodegradability and motion behavior in body fluids, has been employed. Figure 1 shows the fabrication process of such a micromotor, in which Mg microparticles were dispersed on a glass slide precoated with thin layer of poly(vinylpyrrolidinone) (PVP) and coated with degradable polymer poly (lactic-co-glycolic acid) (PLGA) loaded with doxorubicin (DOX) for delivery and release of chemotherapeutic agents. During micromotor propulsion, H_2_, generated by the reaction between Mg and water, plays an important role in enhanced ROS scavenging, thus holding promise for tumor cell treatment [43]. 

Apart from cancer treatment, Mg-based micromotors also have been introduced for capturing and detecting CTCs using the electrochemical method, in which Mg particle surfaces are immobilized with Fe_3_O_4_/P/anti-EpCAM [7]. Another inorganic material with ideal biocompatibility is zinc (Zn), as reported by Zhou et al. As shown in Figure 1B, the tubular micromotor consists of poly(aspartic acid) (PASP) with a thin intermediate Fe layer and internal Zn layer, and the outside surface of the microtubes (negative charge) is further loaded with DOX (positive charge) via electrostatic interaction. Such Zn-based tubular micromotors can be propelled in the presence of gastric acid and permeate the gastric mucus layer, increasing their retention in the stomach [44]. It is well-known that acids like hydrochloric acid react with CaCO_3_ to produce CO_2_ bubbles. As expected, this chemical reaction can be utilized for nano/micromotor propulsion. Recent work reported by Zhang et al. is a typical example. They used yeast cells to synthesize a nano/micromotor by introducing inner- and outer-mineralized CaCO_3_. As shown in Figure 1(Ca), inner nano-CaCO_3_ is generated by the reaction between Ca^2+^ ions (combined with proteins and polysaccharides while entering yeast cells) and CO_3_^2−^ (changed from CO_2_ produced by cell respiration in basic environment), while outer CaCO_3_ is synthesized via the one-pot method using Na_2_CO_3_ and CaCl_2_ to form crystals. This micromotor showed good self-propulsion behavior in gastric fluid (Figure 1(Cb)). Even though the application is related to gastritis therapy instead of cancer treatment, this excellent work provides a new bubble-propelled micromotor with high biocompatibility that, importantly, could be adapted to work in vivo in the stomach [45].

## 3. Bio-Hybrid Nano/Micromotor

**Figure 2:**
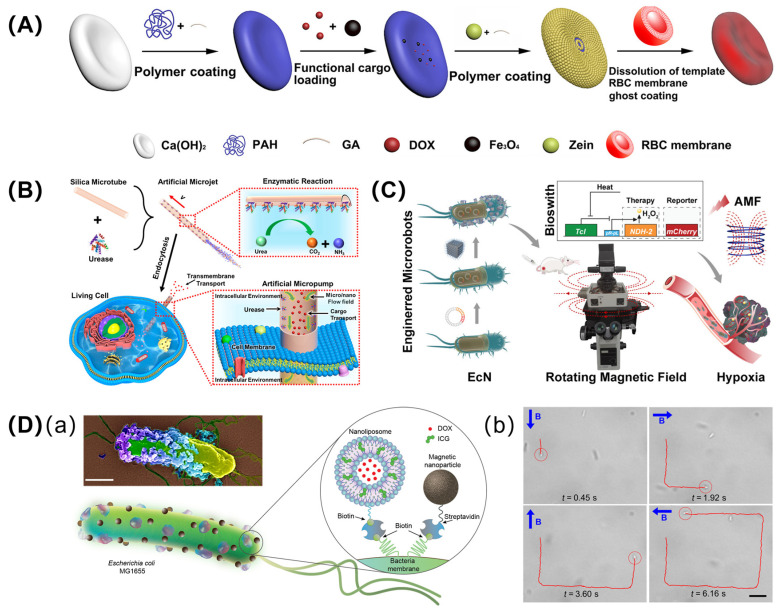
Bio-hybrid nano/micromotor. (**A**) Schematic illustration showing fabrication of RBC-based micromotor. Adapted from [52] with permission, copyright American Chemical Society, Washington, DC, USA, 2022. (**B**) Enzyme-modified tubular micromotor for cargo delivery; adapted from [55] with permission, copyright American Chemical Society, Washington, DC, USA, 2023. (**C**) Engineered hybrid bacteria microrobot for remote collective perception and imaging-guided cancer treatment. Adapted from [61] with permission, American Chemical Society, Washington, DC, USA, 2023. (**D**) Magnetically controlled biohybrid bacterial microrobots for stimulus-responsive cargo delivery: (**a**) Schematic illustration of structure; (**b**) Controlled motion by changing direction of applied magnetic field. Adapted from [63] with permission, copyright American Association for the Advancement of Science, Washington, DC, USA, 2023.

Natural entities such as sperm cells, red blood cells, etc., possess unique properties such as limited immunogenicity, high binding specificity, and the use of bio-safe fuel from the surrounding environment for propulsion. The introduction of biomaterials endows miniaturized actuators with high biocompatibility for working in biological systems [46,47,48]. Natural entity-based nano/micromotors can be categorized into the following four groups. 

(A) Cell membrane. Inspired by nature, the cell membrane, which has unique properties such as immune escape, specific recognition, prolonged circulation time, and high biocompatibility, has attracted the interest of researchers, who coat them onto the surfaces of micromotors. The biological function of natural cell membrane endows the micromotor system with the ability to realize targeted drug delivery or specific binding of bacterial toxins [49,50,51]. One typical example is the use of the red blood cell (RBC) membrane. Hou et al. proposed a cell-mimetic micromotor fabricated using Ca(OH)_2_ microparticles with biconcave discoidal morphology as the template, camouflaged with the RBC membrane. To explore further applications in tumor therapy, Fe_3_O_4_ nanoparticles and DOX(an anticancer drug) were loaded within the wall part of an RBC micromotor for magnetic navigation and tumor therapy (Figure 2A) [52]. Recently, Li et al. introduced a swimming micromotor with clawed geometry by synthesizing sunflower pollen covered with magnetic Fe_3_O_4_ layers as clawed microparticles, followed by the immobilization of an RBC membrane-camouflaged coating. This micromotor proved to have effective magnetic propulsion even against the flow in the rabbit jugular vein. Though no further anticancer experiments were conducted in this work, they provided a promising and safe method of drug delivery in vitro [53]. Another example is the introduction of the cancer cell membrane, which endows gold nano-shell functionalized CaCO_3_ particles with the ability to modulate immune activity, as the coating membrane contains many membrane-bound tumor antigens. The coating layer also enables the micromotor to target corresponding cancer cells due to the homotypic binding of cancer cell membrane [54]. To summarize, these practical examples achieved mimicking of related living cells to endow the nano/micromotor with similar functions, such as biocompatibility or target recognition.

(B) Enzymes. These are mainly proteins that can transform biocompatible fuel into a driving force by catalytic reactions. For instance, by immobilizing urease, a silica-based tubular micromotor is able to catalyze the decomposition of urea, thus generating microfluidic flow via the production of NH_4_^+^ and OH^−^. This micromotor will be endocytosed by the cells and can provide enhanced delivery of anticancer drugs into cells to achieve higher killing efficiency (Figure 2B) [55]. Another example is the modification of glucose oxidase and catalase in which glucose oxidase catalyzes endogenous glucose to produce H_2_O_2_, then the catalase catalyzes the decomposition of H_2_O_2_ (both as produced and natural) for micromotor propulsion. This design shows a synergetic effect for photodynamic-starvation therapy based on the consumption of glucose and the NIR-triggered generation of ^1^O_2_ [56]. In addition, natural platelet cells have also been transformed into biocompatible micromotors, and such cell-based micromotors show propulsion behavior in the presence of urea fuel by the surface modification of urease [57]. An enzyme-based micromotor was also introduced for cancer-related detection, in which catalase was modified on the inner surface of the microtube for propulsion. Based on the decreased motion speed, the researchers realized the bio-sensing of DNA [58].

(C) Bacteria. Bacteria are born with self-swimming ability, which makes them ideal objects for fabricating biohybrid micro-swimmers for drug-delivery purposes [59]. In addition, some bacteria can selectively migrate to the hypoxic regions of solid tumors, which further promotes their development as chemotherapeutic drug carriers [60]. *Escherichia coli* has been incorporated with magnetic nanoparticles for spatial magnetic and hypoxia perception, which provides the collective perception and positive migration ability of microrobots in targeting the tumor microenvironment. Before magnetic modification, bacteria were encoded with bacteria-phage λ repressor cI857 for the triggered expression of the NDH-2 enzyme (respiratory chain enzyme II) and mCherry. Here, the expressed mCherry acts as an internal fluorescence reporter for imaging-guided tracking and actuation along with the NDH-2 enzyme, enhancing anticancer treatment by the upregulation of H_2_O_2_ (Figure 2C) [61]. The gut-friendly bacteria *Lactobacillus rhamnosus* have also been employed for cancer therapy, in which the bacteria were modified with a photoluminescent (Au nanoclusters) and anticancer drug, which showed cytotoxicity to cancer cells [62]. In another work reported by Akolpoglu et al., *Escherichia coli* MG1655 was used as a biological unit to fabricate magnetically controlled microrobots for stimulus-responsive cargo delivery. As shown in Figure 2D, the chosen bacteria expressed biotin attachment peptides, which allowed for the highly efficient modification of nanoliposomes (loaded with photothermal agents and chemotherapeutic molecules) and magnetic nanoparticles (controlled propulsion) via biotin-streptavidin-biotin connections. By applying an external magnetic field, the as-prepared microrobots showed a controlled swimming path (square shaped, as shown in Figure 2(Db)). In another application, bacterial microrobots were also used for the release of anti-cancer drugs by near-infrared light activation [63].

(D) Sperm. Similar to bacteria, sperm cells are natural self-moving microswimmers that can perform complex tasks at microscale [64]. In order to construct a sperm-based anticancer drug-delivery system, man-made tubular microstructures have been designed for the magnetic guidance and release of drug-loaded sperm to an in vitro cultured tumor spheroid [65]. In another work, to prevent the motility of sperm from being affected by surrounding threats such as the specific binding of anti-sperm antibodies, researchers wrapped sperm cells with a zeolitic imidazolate framework-8 (ZIF-8) to maintain their effective propulsion [66].

## 4. Ultrasound Waves for Propulsion

**Figure 3:**
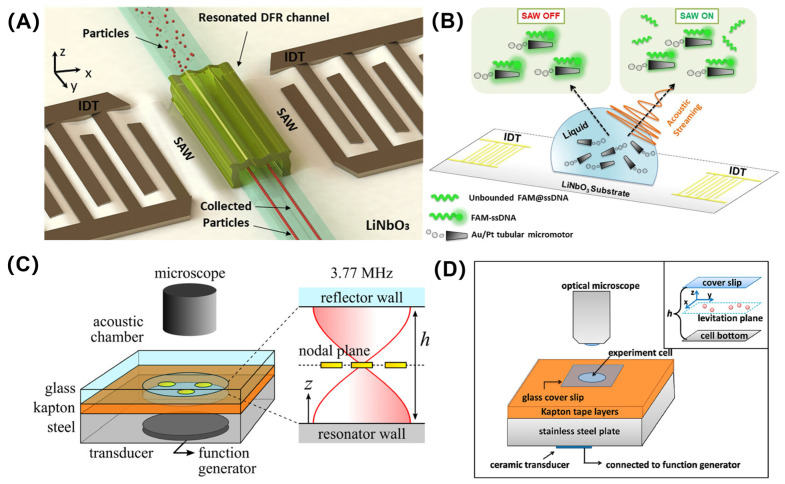
Topical devices used to generate ultrasound for nano/micromotor manipulation. (**A**) Schematic illustration showing surface acoustic wave tweezer for collection of submicron particles and human blood cells. Adapted from [70] with permission, copyright American Chemical Society, Washington, DC, USA, 2023. (**B**) Schematic illustration showing surface acoustic wave tweezer to remove molecules unbound to micromotors, thus lowering detection limit of cancer-related biomarker miRNA-21. Adapted from [71] with permission, copyright American Chemical Society, Washington, DC, USA, 2021. (**C**,**D**) Schematic illustration showing two tailor-made chambers for reflecting ultrasound waves when using standing ultrasonic waves for nano/micromotor propulsion. Adapted from [72,73] with permission, copyright American Chemical Society, Washington, DC, USA, 2018, 2012.

Ultrasound, which uses sound waves created by vibrating objects and is a type of mechanical wave, is not only widely used for medical imaging, but it also can serve as a biocompatible propulsion mode for nano/micromotors. Compared to other energy inputs, ultrasound has wide clinical use (for imaging), and its ability to penetrate deeply through tissue has been well-proved. By using ultrasound as the energy input, nano/micromotors can avoid using toxic fuel and, thus, be highly biocompatible. Ultrasound is also currently being widely used for nano/micromotor propulsion [67,68,69]. Both surface acoustic waves (SAWs) and ultrasonic standing waves (USWs) can be used for nano/micromotor manipulation. SAWs usually use piezoelectric ceramics (such as lithium niobate, LiNbO_3_) as the substrate with interdigital transducers (IDTs) on the surface (Figure 3A,B) [70,71], and for USWs, researchers tend to carry out nano/micromotor propulsion in a tailor-made chamber (made from Kapton tape or PDMS) for ultrasound wave reflection and piezoelectric ceramics stuck to the chamber for ultrasound wave generation (Figure 3C,D) [72,73].

**Figure 4:**
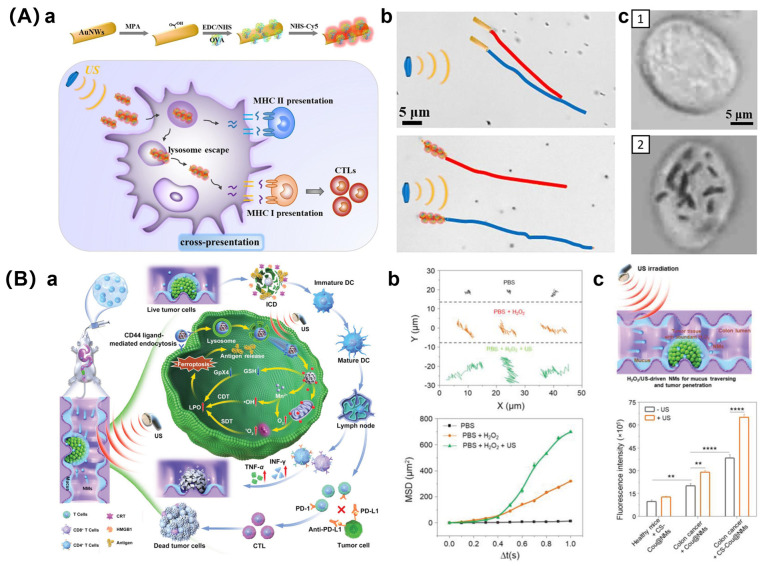
Ultrasound as biocompatible energy input to drive nano/micromotors for cancer-related treatment. (**A**) Ultrasound-propelled gold nanowire nanomotors for improved antigen (ovalbumin) cross presentation and cellular immunity: (**a**) Concept of this work; (**b**) Time-lapse images of tracking trajectories of nanomotor; and (**c**) DC 2.4 cells treated with free ovalbumin and ovalbumin-modified nanomotors. Adapted from [74] with permission, copyright Elsevier Ltd., Amsterdam, The Netherlands, 2021. (**B**) Ultrasound-propelled nanomotors with multiple functions, including colonic mucus-traversing ability, deep tumor tissue penetration, and anti-tumor immunity: (**a**) Schematic illustration of this work; (**b**) Motion trajectories of nanomotor under different propulsion modes and corresponding mean square displacement (MSD); and (**c**) Schematic illustration of mucus-traversing and tumor penetration of nanomotors propelled by microbubbles and ultrasound, and fluorescence intensity showing differences with and without ultrasound irradiation in healthy control mice and mice with orthotopic colon tumors treated with oral administration of coumarin-6 (fluorescence probe)-modified nanomotors (*n* = 3, ** *p* < 0.001, and **** *p* < 0.0001; two-way ANOVA with Tukey’s test). Adapted from [75] with permission, copyright Wiley Ltd., Hoboken, NJ, USA, 2022.

Wang et al. synthesized nanomotors using gold nanowires (AuNWs) modified with ovalbumin (OVA), which act as model protein antigen. The nanowires are also propelled by ultrasound and realized antigen delivery (Figure 4(Aa)). Such acoustically active AuNWs@OVA nanomotors retain high speed (only a little decrease from average 90 to 61 μm/s), even coating the surfaces with protein (Figure 4(Ab)) and show the ability to enter single cells without disrupting the integrity of the cell (Figure 4(Ac)). This novel design provides a new strategy for solving the challenge of the degradation of internalized exogenous antigens in lysosomes while using the vaccine. These ultrasound-powered nanomotors help in the process of antigen cross presentation and cellular immunity (with upregulation of MHC I and MHC II-related molecule expression), which are critical components of the immunological effect of therapeutic vaccines for tumors or viral diseases [74]. Cao et al. synthesized mesoporous manganese oxide (MnO_x_) using a water/oil emulsion reaction at room temperature, followed by the loading of indocyanine green derivatives (IDs) for mitochondrial targeting, chondroitin sulfate (CS) for specific drug delivery in colon tumors, and regenerated silk fibroin (RSF) to form macromolecular layers for ID release after pH/reactive oxygen species/glutathione simulation (Figure 4(Ba)). These nanomotors were dual-propelled by oxygen bubbles and ultrasound, and they showed apparently higher average speed than the single-engine mode (Figure 4(Bb)). The penetration of nanotherapeutics into tumor tissue is critical to achieve efficient anti-tumor activity, and the results show that the nanomotors could traverse the colonic mucus layer and penetrate into internal colon tumor tissue with the aid of ultrasound. The nanomotors would also be internalized with partial epithelial cells after oral administration, and ultrasound irradiation would slightly help them penetrate into healthy colon tissue (Figure 4(Bc)). The release of Mn ion is helpful in the decomposition of overproduced H_2_O_2_ in the tumor microenvironment to produce ·OH and O_2_ (Fenton-like reaction). The produced ·OH subsequently induces chemodynamic therapy (CDT), and O_2_ strengthens the sonodynamic therapy (SDT) effect as well [75].

## 5. Electromagnetic Wave (Light)-Based Propulsion

Electromagnetic waves such as light provide a clean, harmless, and noninvasive propulsion mode, making it a versatile and powerful candidate for driving nano/micromotors that are highly biocompatible. In addition, by using electromagnetic waves for this purpose, researchers can easily control the speed and direction of nano/micromotors, and no complex or special equipment is required compared to other propulsion modes such as magnetic or ultrasound waves [76,77,78]. Different wavelengths of electromagnetic waves, ranging from ultraviolet (UV) to visible (VIS) to near-infrared (NIR) light [79,80,81], as well as X-rays [82], have been used for nano/micromotor propulsion. In addition, materials such as Ag_3_PO_4_ [83], BiVO_4_ [84], Ag [85], Cu@MoS_2_ [86], carbon nitride (f-C_3_N_4_) [87], TiO_2_ [88], BiOI/AgI/Fe_3_O_4_/Au [89], ZnO/Pt [90], and Cu_2_O@CdSe [91] have been smartly designed and utilized for the construction of nano/micromotors driven by electromagnetic waves. The mechanism behind such propulsion is usually complex and can include electrophoretic and diffusiophoretic effects or the generation of an interfacial tension or temperature gradient [77,80,92].

**Figure 5:**
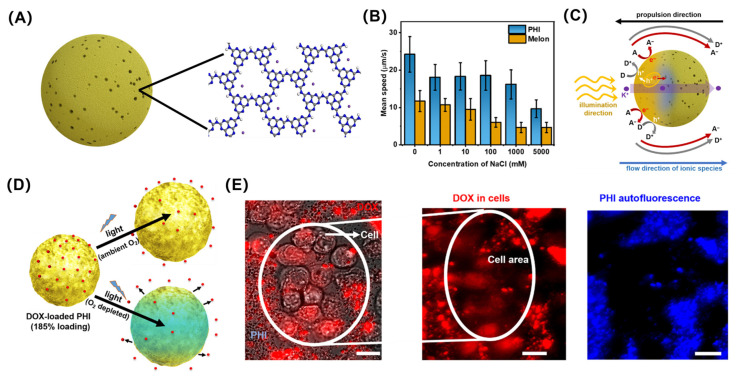
Light-propelled carbon nitride-based microswimmers used to overcome high-ion tolerance, which remains a challenge in light-propelled nano/micromotors. (**A**) Schematic illustration of PHI micromotor and structure of PHI macromolecules. (**B**) Mean speed of PHI and one-dimensional (1D) CNx (melon)-based micromotors with an increased NaCl concentration via 385 nm irradiation. (**C**) Schematic illustration of mechanism for light propulsion and related ionic tolerance of PHI micromotor. (**D**) Schematic illustration of PHI micromotors loaded with DOX showing drug-release behavior in response to ambient and hypoxic conditions. (**E**) Left: optical microscopy and fluorescence images of cancer cells with DOX-loaded PHI micromotors under light/415 nm irradiation and released drug consumed by cells with emission at 595 nm fluorescence; right: PHI autofluorescence revealing that PHI micromotors adhered to cancer cells after some DOX release. Adapted from [96] with permission, copyright American Association for the Advancement of Science, Washington, DC, USA, 2022.

Currently, using light-propelled nano/micromotors for cancer treatment is a research hot topic. For instance, Xing et al. fabricated jellyfish-like mesoporous carbon nanomotors integrated with single-atom copper (Cu-JMCNs) propelled by the thermophoretic effect after NIR light irradiation. By integrating single Cu atoms, H_2_O_2_ was catalyzed to produce toxic hydroxyl radicals for chemodynamic therapy, and an NIR-triggered motion of the nanomotor improved cellular uptake and tumor penetration [93]. With light propulsion, one major drawback is the inability to be propelled in solution with high ionic strength because existing ions would restrain the formation of concentration gradients, which would contribute to the self-electrophoresis or self-diffusiophoresis effect around the nano/micromotors [94,95]. To overcome the above challenge, Sridhar et al. employed two-dimensional (2D) poly(heptazine imide) (PHI) carbon nitride to build light-propelled micromotors (Figure 5A). Thanks to the proper interaction between the textural and structural nanoporosity and optoionic properties of particles, the proposed microswimmer achieved propulsion in a highly ionic solution (Figure 5B). Compared to traditional one-dimensional (1D) CN_x_, the PHI show both higher hydrogen evolution activity and the ability to store light-induced electrons. As shown in Figure 5C, the authors supposed that the mechanism of the light-simulated microswimmer motion mainly came from asymmetric illumination and photocatalysis, which caused ion flow around and through the materials. The cations move across the pores of the material to counteract Debye layer collapse, thus contributing to the ionic tolerance. In addition, a pseudocapacitive photo-charging effect occurs in the materials to further strengthen ionic tolerance with respect to 1D CN_x_. The light-propelled PHI micromotors were further loaded with doxorubicin (DOX) and showed stimulus-responsive drug release when triggered by hypoxia, pH, and light (Figure 5D,E) [96].

## 6. Magnetic Propulsion

Nano/micromotors propelled by an external magnetic field have advantages including being fuel-free and having precise and controllable motion [97,98]. In this propulsion mode, nano/micromotors seem to be simply propelled by the external magnetic field, yet they also involve energy conversion during the self-propulsion process. Indeed, these nano/micromotors have potential magnetic energy that is relative to the outside magnetic source; during propulsion, the potential magnetic energy is transformed into kinetic energy. Various techniques have been proposed to enable the magnetic response of nano/micromotors for directional or propulsion purposes, such as the chemical synthesis of magnetic particles followed by the encapsulation or surface modification [99,100], physical vapor deposition [101,102], electrochemical deposition [103], 3D printing using direct laser writing [104,105], and the combination of microfluidic droplet printing and wettability-induced drawing photolithography [106]. For magnetic guidance or propulsion, various devices such as Helmholtz coil [107], Maxwell coil [108], and saddle coil [109] have been designed to provide a uniform or gradient magnetic field.

**Figure 6:**
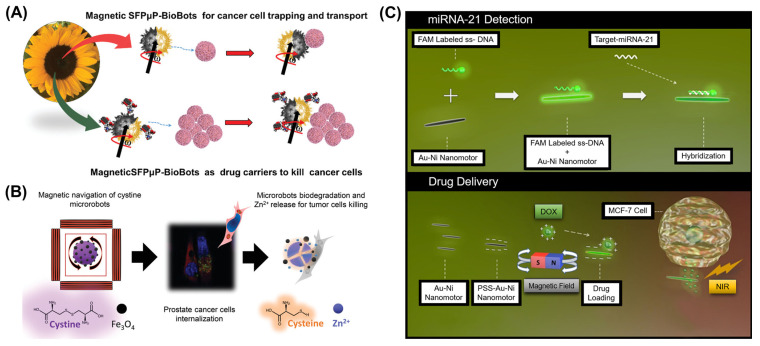
Magnetic field as biocompatible energy input for nano/micromotor propulsion. (**A**) Schematic illustration showing magnetic micromotor fabricated by sputtering natural sunflower pollen with Au, Co, and Au, which was used as a drug carrier to attack and kill cancer cells. Pink balls represent cancer cells. Adapted from [110] with permission, copyright Wiley Ltd., Hoboken, NJ, USA, 2023. (**B**) Schematic illustration of magnetically propelled cystine micromotor for treatment of prostate cancer. Adapted from [111], permission not needed for this open access article, copyright Wiley Ltd., Hoboken, NJ, USA, 2023. (**C**) Top image shows magnetically propelled Au-Ni nanowires for cancer biomarker detection; bottom image shows chemotherapeutic drug delivery. Adapted from [112] with permission, copyright American Chemical Society, Washington, DC, USA, 2021.

For cancer treatment, Mayorga-Martinez et al. used sunflower pollen deposited on thin-film metal layers (including Au, Co, and Au) on one side of the microsphere, thus endowing it with magnetic response ability for micromotor propulsion. This type of micromotor shows good performance in attracting cancer cells due to the electrostatic interactions between them and can be loaded with DOX to kill cancer cells (Figure 6A) [110]. In another work, researchers introduced magnetically actuated cystine micromotors by the zinc-mediated self-assembly of cystine and the encapsulation of Fe_3_O_4_ nanoparticles during the synthesis process. This cystine micromotor could be efficiently internalized in late endosome/phagolysosome compartments before Zn^2+^ ions are released for killing tumor cells after the bio-enzymatic degradation of micromotors due to broken disulfide bonds (Figure 6B) [111]. For cancer detection, magnetically propelled gold-nickel nanowires were fabricated by template electrochemical deposition and used for rapid/sensitive sensing of the cancer biomarker microRNA-21. The fluorescent dye-labeled ssDNA probe was first immobilized on a nanomotor, and its target-miRNA-21 present in the solution was hybridized with the ssDNA probe on the nanomotor surface, thus decreasing the fluorescence intensity and motion speed related to the target biomarker concentration. Au-Ni nanomotors were further physisorption loaded with DOX via the hydrophobic interaction after being modified with poly(sodium 4-styrenesulfonate) (PSS) to provide active chemical groups for DOX interaction. Related results show a pH-dependent drug release of DOX-loaded nanomotors, as well as the magnetic guidance of the nanomotors on MCF-7 cells, with efficient and controlled drug delivery (Figure 6C) [112].

## 7. Dual-Propelled Nano/Micromotors

Integrating two different propulsion modes into one nano/micromotor, also called a dual-propelled nano/micromotor, can realize more flexible and efficient movement. By employing two engines, nano/micromotors can carry out cargo transportation in more complex situations, and it is more convenient to control their speed and direction. The dual-propulsion mode can include bubble (chemical)/light [113], magnetic/bubble (biocatalytic enzyme) [114], light/magnetic [115], bubble (chemical)/ultrasound [116], ultrasound/magnetic [117], or ultrasound/light [118], which have also been used for cancer-related treatments or detection. For instance, a combination of enzyme-based chemical energy and magnetic field energy has been developed to drive micromotors for synergistic anticancer therapy. Here, the enzymatic-based decomposition of glucose leads to self-propulsion, and the magnetic energy provides controllable movement [31]. In a photodynamic-based cancer therapy strategy, ultrasound was employed as an efficient and bio-safe energy for the propulsion of blood cell-mimicking (RBCM) micromotors. By integrating Fe_3_O_4_ NPs, it is possible to orient the motion of RBCM micromotors under an external magnetic field [119]. The hydrothermal method was utilized to synthesize dendrite-shaped microrobots that exhibited dual light/magnetic propulsion. These micromotors showed negative phototaxis due to the self-diffusiophoresis effect under light irradiation, while the external magnetic field endowed them with rolling-motion behavior. By exposure to light along with H_2_O_2_, such micromotors could realize on-site ROS generation to deplete GSH and enhance PDT efficiency for prostate cancer therapy [120].

Dual-propelled nano/micromotors not only have been well-designed for cancer treatment as described above, but they also have played an important role in related disease detection. For instance, Báezet et al. synthesized tubular micromotors propelled by chemical catalytic and magnetic energy for gastric cancer biomarker detection [121]. Ren designed nanomotors propelled by magnetism and bubbles, in which γ-Fe_2_O_3_ nanorods were used to coat the catalase. The modification of folic acid (FA) and hyaluronic acid (HA) carbon dots endowed the nanomotor with target recognition ability. This nanomotor showed the ability to capture specific circulating tumor cells, provide imaging (fluorescence from carbon dots), and achieve quantitative detection due to the modification of the recognition elements (FA and HA), thus providing a new possibility for cancer detection [122].

## 8. Microswarm

In order to use nano/micromotors for cancer treatment or imaging, they can be designed as individual units to perform the task or as microswarms. Microswarm, which refers to the concept that large numbers of micro/nanoparticles can work together cooperatively, seems to not be directly related to biocompatibility. Yet it represents an important and effective tool for further reducing nano/micromotor toxicity, thus promoting clinical applications. For cancer therapy, generally efficacy and safety are two critical aspects when evaluating a new drug. Thus, researchers need to design the correct dosage by balancing the benefits and harms to achieve the desired therapeutic efficacy. A higher curative effect means a lower dosage, which may reduce the side effects of cancer treatment. Similarly, improving the cancer treatment efficacy of drug-loaded nano/micromotors would indirectly affect their biosafety [123,124,125]. For drug delivery, the cooperative behavior of nano/micromotors would enable the use of larger doses of loading drugs for delivery compared to individual agents, therefore enhancing therapeutic efficacy [126,127]. A recent published review by Sun’s group also pointed out that the swarm behavior of nano/micromotors provides possibilities for drug delivery with longer retention time, which in turn would allow significantly lower doses for higher biocompatibility [128]. For medical imaging, researchers have also proved that with ultrasound imaging, introducing microswarms acting as imaging contrast would reduce the minimal required dose of nanoparticles, indicating a lower dosage for potentially higher biocompatibility [129,130].

**Figure 7:**
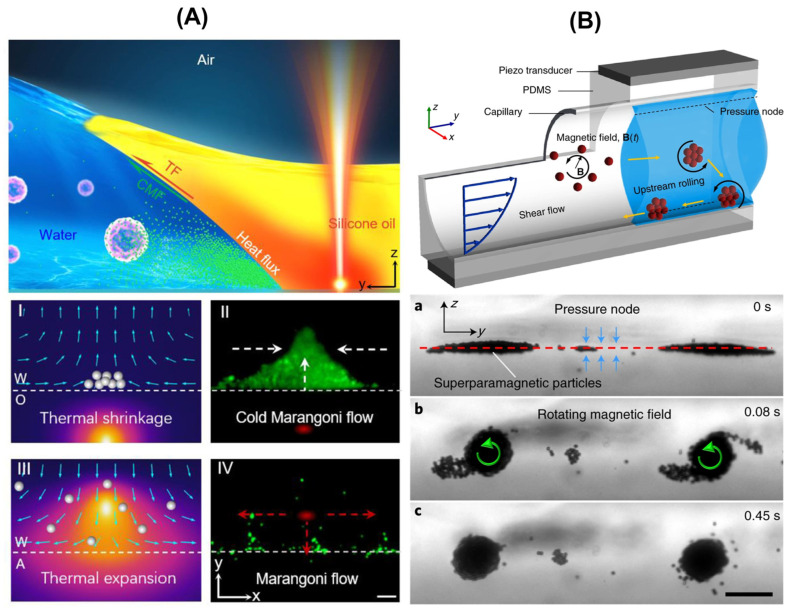
Smart designs for in vivo application of microswarms. (**A**) Schematic illustration showing cold Marangoni flow generated at water/oil interface after light irradiation and comparison between cold (**I**,**II**) and conventional (**III**,**IV**) Marangoni flow; (**I**,**III**) show simulated heat distribution and flow field distribution; arrows indicate the streamlines; (**II**,**IV**) show experimental results of microswarm formation. Adapted from [131] with permission, copyright Wiley Ltd., Hoboken, NJ, USA, 2023. (**B**) Schematic illustration shows concept of acousto-magnetic propelled microswarm against the flow, and microscopy images show formation of microswarms under the outside rotating magnetic field and ultrasound field at 0 s (**a**), 0.08 s (**b**), 0.45 s (**c**). Adapted from [132] with permission, copyright Springer Nature, New York, NY, USA, 2023.

A conventional microswarm manipulated by a photothermal-based mechanism mainly relies on a light-simulated temperature gradient or interfacial tension gradient, thus producing fluid flow for object motion. However, this strategy may suffer from the heat damage generated by light irradiation. To address this issue, Shi et al. developed cold Marangoni flow for microswarm actuation, which allows for the targeted gene delivery but avoids heat damage to targeted cells. They manipulated the microswarm in a water droplet surrounded by silicone oil, and by the irradiation of infrared light, a temperature gradient was produced at the water–silicone oil interface due to the strong light absorption of the oil and very weak light absorption of water, thus further causing the interfacial tension gradient to contribute to the strong Marangoni flow. Importantly, in this work, they found no significant heat transfer in the water due to the strong convection near the water–silicone oil interface in the special reverse Marangoni flow. It should be noted that in a conventional case that occurs near the water–air interface, the Marangoni flow moves from the higher temperature area to the surrounding area. Thus, here they propose a biocompatible strategy for microswarm manipulation compared to the conventional method that is normally accompanied by photothermal damage (Figure 7A) [131]. Apart from the biocompatibility of the nano/micromotor and the fuel or other energy input that need to be considered, another challenge that restricts the use of these micro/nanodevices in in vitro and in vivo applications is the complex outside microenvironment. For instance, the flow inside a blood vessel would make such micro/nanoparticles follow the stream. In turn, they are unable to be controlled. To address this, Ahmed et al. proposed the use of an acousto-magnetic microswarm to propel against such flow. They used an outside rotating magnetic field to turn superparamagnetic particles (about 3 μm) into a microswarm, followed by an ultrasound field to guide the assembled microswarm toward the capillary wall, thus achieving upstream rolling and rheotaxis. Here, both energy inputs (magnetic and ultrasound) are highly biocompatibility and can penetrate deeply into the body, providing a new strategy for delivering targets to hard-to-reach sites and go against the blood flow, which is important for future clinical applications (Figure 7B) [132].

## 9. In Vivo Nano/Micromotor Visualization

**Figure 8:**
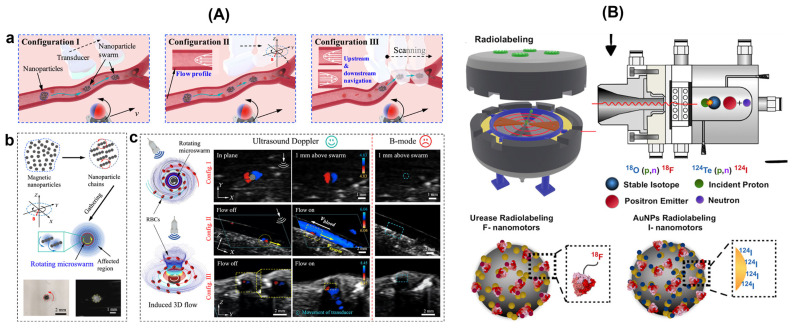
Medical imaging technology for microswarm tracking. (**A**) (**a**) Shows schematic illustration of magnetic microswarm navigation in blood vessels; (**b**) Is formation process of a microswarm; (**c**) Is Doppler ultrasound signal around rotating microswarm in blood, showing Doppler signals in stagnant and flowing blood near microswarm, as well as tracking of microswarm in real time. Adapted from [133], permission not needed for this open-access article, copyright American Association for the Advancement of Science, Washington, DC, USA, 2023. (**B**) Schematic illustration showing ^18^F- and ^124^I-radio-labeled nanomotors that can be tracked by positron emission tomography (PET). Adapted from [134] with permission, copyright American Association for the Advancement of Science, Washington, DC, USA, 2023.

When using nano/micromotors for targeted drug delivery in vivo, it is difficult to track them in real time because visible light cannot penetrate the tissues. In consideration of this, medical-imaging technologies based on different mechanisms such as ultrasound, positron emission tomography, or magnetic resonance have been introduced. Ultrasound not only can drive nano/micromotors, as mentioned above, but it also is a powerful imaging technique based on the reflection of mechanical waves. Wang et al. realized the manipulation of a magnetic microswarm near the boundary of vessels, with the ability to navigate upstream and downstream even in flowing conditions due to the reduced drag force from blood flow and strong interactions between nanoparticles. When ultrasound waves are produced and travel to the microswarm, the Doppler effect occurs, which can subsequently be detected by a Doppler ultrasound imaging device. Thus, the position of the microswarm can be tracked in real time (Figure 8A) [133]. It should be noted that the spatial resolution of ultrasound imaging technology is at the scale of millimeters, which is insufficient for imaging individual micro/nanorobots, which are typically at micrometer scale. Thus, it is necessary to use a microswarm with cooperation behavior. Another medical imaging technology, positron emission tomography (PET), has also been used to track microswarms, in which short-lived radioactive substances, including ^124^I on gold nanoparticles or ^18^F-labeled urease, were labeled on nanomotors with an enzyme-based engine that showed swarm behavior. During in vivo experiments, the radio-labeled nanomotors underwent positive beta decay and emitted positrons to further interact with ordinary electrons, followed by particle annihilation, γ-ray emission, and, finally, ray detection. Due to the highly efficient tissue penetration of γ-rays, researchers determined by analysis that the biodistribution of nanomotors could be realized after being injected intravenously in female mice (Figure 8B) [134].

Magnetic resonance imaging (MRI) has also been utilized for tracking or imaging purposes. In one study, Fe_5_C_2_@Fe_3_O_4_ nanoparticles were fabricated for both magnetic targeting and T2-weighted MRI and showed great potential for imaging-guided therapy [135]. The same group recently developed FeO@mSiO_2_/Au-CAT Janus nanorobots for enhanced tumor penetration and therapy. With the high spatiotemporal resolution and deep penetration of MRI, the migration of nanorobots can be monitored in a non-invasive way in real time [136].

Table 1 provides some typical examples of the use of biocompatible energy for nano/micromotor propulsion.

## 10. Conclusions and Perspectives

Owing to rapid advances in nanotechnology and material chemistry, nano/micromotors show great potential for cancer detection and treatment. Yet biotoxicity usually restricts them from being further employed in clinical applications. Currently, the use of nano/micromotors for cancer therapy is still in the early research stage. However, some in vivo applications have been attempted in animal experiments. While we focused on the use of nano/micromotors for disease therapy from a wider perspective, we found that Zn-based [44] and Mg-based [145] micromotors have been tested in animal stomachs. Slippery micropropellers have been used to penetrate the vitreous body of porcine eyes [146], magnetically controlled microrobots with clawed geometry and red blood cell membrane coating have been injected into the blood vessels of rabbits [53], and Doppler ultrasound has been used for tracking magnetic microswarms in porcine coronary artery ex vivo [133]. All of these examples indicate that nano/micromotors are a promising technology for cancer diagnosis and therapy. In this review, we discussed the biocompatibility aspect of recent developments of nano/micromotors to show how researchers have tried to reduce biotoxicity by introducing bio-safe propulsion modes, integrating biomembranes or living micro-entities (bacteria or sperm), etc. Even though researchers are trying their best to conquer this bottleneck, more breakthroughs are needed to promote the advancement of this area.

Recent advances have shown the great success of fabricating biocompatible bio-hybrid nano/micromotors by introducing active proteins or living micro-entities (cell membrane, enzyme, sperm, microorganism, etc.). Although most of them exhibited biosafety in cell viability or hemolysis tests, their immune response also needs to be considered before they can be used in further applications. Using ultrasound as a bio-friendly energy input shows advantages such as noninvasive and deep-tissue penetration. However, a medium to conduct or propagate its energy is still needed, and in some instances, appropriate cavity structures for wave reflection are required. Light also has been developed as a noninvasive, clean, and harmless propulsion mode, although a critical drawback that restricts further in vivo application is the opaque quality of tissues. Therefore, more special devices such as optical fiber-based devices should be designed to induce the external light inside the body [147]. Magnetic propulsion does not require an energy propagation medium, reflection cavity, or tissue transparency, and nano/micromotors can be controlled easily by changing the direction of the external magnetic field, which makes it a promising candidate for in vivo applications. Yet this modality needs the nano/micromotors to have a good response to the external magnetic field to overcome obstacles such as blood flow or other complex situations inside the body. Therefore, future efforts should focus on developing new magnetic materials with low toxicity but good magnetic response. Apart from that, the degradability of the materials, the size effect of the nano/micromotor, and comparisons between individual nano/micromotors all deserve further investigation.

Another critical point is that when designing biocompatible nano/micromotors for cancer diagnosis, various strategies such as fluorescence-based strategies, colorimetric methods, electrochemistry, SERS-based biosensing, etc., have been integrated for analyte detection. Particularly, speed change is a unique feature of nano/micromotors, in that the speed can be reduced or increased after meeting specific analytes. Such change could easily be observed and recorded by normal light microscopy (visual signals), which provides a new strategy for bio-sensing. Future work should also focus on designing related cancer-diagnosis methods using motion-based sensing [58,148,149]. In the design of biosensors for bio-molecular detection, the motion-based strategy is a unique and effective method that originated from the self-propulsion behavior of nano/micromotors [130,150]. Yet this detection strategy mainly depends on the tracking of individual nano/micromotors, which does not work for on-site detection inside the opaque body. Therefore, innovations in the tracking of separate nano/micromotors would provide new possibilities for on-site disease diagnosis by using motion-based strategies.

In addition, for cancer diagnosis, micro-mixing, along with the enhanced mass transfer produced by micromotor propulsion, has been utilized to enhance biosensing efficiency [139,151,152,153]. Still, nano/micromotors with different motion behaviors (microswarm versus individuals) have not been investigated. A recent work [154] shows that the collective behavior of microswarms would be helpful in generating high convection and micro-mixing for enhanced mass transfer. Even though researchers are focusing on using microswarms for water remediation, it is important to investigate whether microswarms could help enhance biosensing efficiency compared to the micro-mixing effect caused by individual nano/micromotors. Secondly, even though ultrasound imaging is a powerful tool for dynamic tracking of miniaturized actuators inside the body, only microswarms can currently be detected and tracked since there is limited spatial resolution due to the diffraction limit and related wavelength of ultrasound waves [155]. As a result, understanding the dynamic position of an individual nano/micromotor inside the body remains a challenge at the moment.

## Figures and Tables

**Table 1:** Some typical nano/micromotors with biocompatible modes designed for cancer diagnosis and treatment.

Biocompatible Driving Mode	Micromotor Design		Application	Ref.
Inorganic chemical-based energy				
Mg	Mg microparticles coated with PLGA and DOX	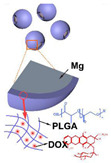	Inhibit tumor growth/deliver H_2_ (produced by Mg) and chemotherapeutic drug (DOX)	[43]
Mg	Mg particle surface immobilized with Fe_3_O_4_/polydopamine/anti-EpCAM	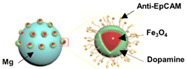	Capture and detect CTCs	[7]
Zn	DOX-loaded poly(aspartic acid)/Fe-Zn microrocket	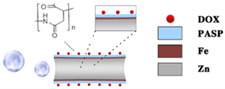	Targeted drug delivery	[44]
CaCO_3_	Yeast-based Cur@CaY-robot with inner CaCO_3_ for encapsulating curcumin, outside CaCO_3_ for propulsion	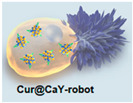	Improve drug agent accumulation, release Ca^2+^ for synergistic repair of gastric motility in mice with gastritis	[45]
Bio-hybrid				
Enzyme/ urease	Urease immobilized asymmetrically onto surface of platelet cells	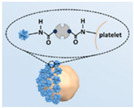	Targeted anticancer drug (DOX) delivery in vitro	[57]
Enzyme/ catalase	Catalase modified on inner surface of microtube	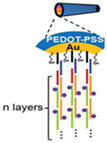	Decrease motion speed for DNA sensing	[58]
Sperm	Magnetic microcap/sperm loaded with anticancer drug	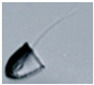	Patient-specific 3D ovarian cancer treatment, drug loading, and delivery	[137]
Magnetotactic bacteria	Photosensitizer chlorin e6 (Ce6) incorporated into *M. magneticum* strain AMB-1	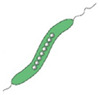 AMB-1/Ce6	Targeted tumor therapy	[138]
Magnetic field				
Rotating magnetic field	Magnetic helical hydrogel (PVA/MA/Fe_3_O_4_)	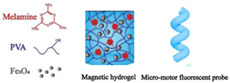	Tumor DNA detection	[139]
Rotating magnetic field	Magnetic photonic-crystal microrobots (pH-responsive hydrogel microspheres encapsulated with Fe_3_O_4_ NPs)	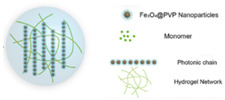	pH mapping of microenvironment, drug delivery to specific tumor site, visual pH detection, DOX loading, and pH response to drug release	[140]
Rotating magnetic field	Silica-coated magnetic rod with Ag NPs grown on surface	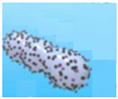	Enhance sensing sensitivity of surface-enhanced Raman scattering (SERS)	[8]
Gradient magnetic field	Bacteria loaded with magnetic NPs and nanoliposomes	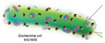	Liposomal drug delivery (DOX) and NIR response release	[63]
Ultrasound waves				
Standing acoustic waves	Graphene oxide-coated gold nanowire motors functionalized with fluorescein-labeled DNA aptamer	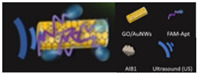	Sense AlB1 in breast cancer cells by fluorescence OFF-ON strategy	[141]
Surface acoustic waves (SAWs)	Bimetallic Au/Pt tubular micromotor	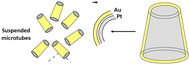	Fluorescence detection of miRNA-21	[71]
Light irradiation				
Optical tweezers	Upconversion fluorescence micromotor (lanthanide doped microrods)	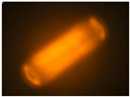	Determine abnormal temperature of cells, deliver DOX-loaded mesoporous silica to vicinity of cancer cells	[142]
NIR (808 nm)	Au/Cu micromotor swarm	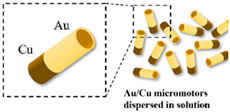	Synergistic photothermal strategy to eradicate tumors	[143]
Dual-propelled micromotor				
Magnetic/ electric	Janus microsphere structure formed by coating fluorescent polystyrene sphere with Cr, Ni, and Au	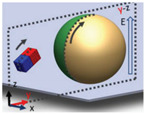	Identify apoptotic cells based on dielectrophoretic difference and use for cargo transport	[97]
Light/gas	DOX-loaded CaCO_3_/Au Janus micromotor	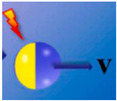	Overcome sequential biological barriers during tumor-targeted drug delivery	[144]
Microswarm				
Light	Microswarm actuation in a water droplet surrounded by silicone oil (cold Marangoni flow)	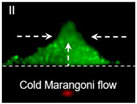	Cold Marangoni flow (avoiding heat damage) for microswarm manipulation and targeted gene delivery	[131]
Ultrasound/ magnetic	Magnetic field for forming microswarm and ultrasound for guidance	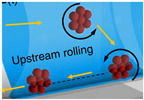	Acousto-magnetic microswarm to propel against flow	[132]

Images are adapted from [7,8,43,44,45,57,58,63,71,97,119,120,137,138,139,140,141,142,143,144] with permission, copyright Elsevier Ltd., Wiley Ltd., American Chemical Society, Royal Society of Chemistry, American Association for the Advancement of Science, and Springer Nature.
